# MicroRNA Sequencing Reveals the Effect of Different Levels of Non-Fibrous Carbohydrate/Neutral Detergent Fiber on Rumen Development in Calves

**DOI:** 10.3390/ani9080496

**Published:** 2019-07-28

**Authors:** Mingming Xue, Kejun Wang, Ansi Wang, Ruiting Li, Yadong Wang, Shuaijie Sun, Duo Yan, Guohua Song, Huifen Xu, Guirong Sun, Ming Li

**Affiliations:** College of Animal Science and Veterinary Medicine, Henan Agricultural University, Zhengzhou 450046, China

**Keywords:** calf, rumen, papilla, miRNA

## Abstract

**Simple Summary:**

By histological sectioning and staining of rumen tissues from calves fed with a high or low ratio of non-fibrous carbohydrate/neutral detergent fiber diet, we found that the length and width of papillae were significantly affected by the ratio. From microRNA expression analysis we found cell proliferation, differentiation, physical and nutrient stimuli processes participate in the development of the rumen. In addition, bta-miR-128 was found to affect rumen development by negatively regulating *PPARG* and *SLC16A1*. Our findings provided an important resource for the continuing study of rumen development and absorption.

**Abstract:**

Rumen development in calves is affected by many factors, including dietary composition. MicroRNAs (miRNAs) are known to function in the development of the rumen in cattle, what is not known is how these miRNAs function in rumen development of calves fed with high and low ratios of non-fibrous carbohydrate (NFC)/neutral detergent fiber (NDF). A total of six healthy Charolais hybrids bull calves of similar weight were divided into two groups; three calves were fed a mixed diet with NFC/NDF = 1.35 (H group), and three were fed a mixed diet with NFC/NDF = 0.80 (L group). After 105 days on the diet, calves were sacrificed and rumen tissues were collected. Tissues were subjected to histological observation and miRNA expression analysis. Functional enrichment analysis was conducted on the target genes of the miRNAs. Targeting and regulatory relationships were verified by luciferase reporter assay and quantitative PCR (qPCR). We found that the length of rumen papilla in the L group was significantly greater than that in the H group, while the width of rumen papilla in H group was significantly greater than that that in L group. We identified 896 miRNAs; 540 known miRNAs, and 356 novel predicted miRNAs. After statistical testing, we identified 24 differentially expressed miRNAs (DEmiRNAs). miRNA-mRNA-cluster network analysis and literature reviews revealed that cell proliferation, differentiation, physical and nutrient stimuli processes participate in rumen development under different NFC/NDF levels. The regulatory relationships between three DEmiRNAs and five target genes were verified by examining the levels of expression. The binding sites on bta-miR-128 for the peroxisome proliferator activated receptor gamma (*PPARG*) and solute carrier family 16 member 1 (*SLC16A1)* genes were investigated using a dual luciferase assay. The results of this study provide insight into the role of miRNAs in rumen development in calves under different NFC/NDF levels.

## 1. Introduction

The rumen is the primary site for fermentation in ruminant animals as well as an important site for nutrient absorption, digestion, and metabolism. Digestion and metabolism mainly involve the degradation of fiber and the absorption of volatile fatty acids by the rumen epithelium. Rumen epithelial morphology and development is affected by the feed quality and particle size [1,2] which obviously has an affect on the animal health and growth. Several publications have suggested that miRNAs play a crucial role in regulating the rumen development during bovine embryonic development [3,4,5,6]. However, the effect of different NFC/NDF levels on miRNAs involved in the rumen development process in calves is unclear.

MiRNAs are a class of non-coding single-stranded RNA molecules, approximately 22 nucleotides (nt) in length, that are involved in post-transcriptional regulation of gene expression in plants and animals, including early development [7], cell proliferation, apoptosis, cell death [8], cell differentiation [9], and fat metabolism [10]. In broad terms miRNAs degrade their target mRNA or inhibit its translation, although they function in many other ways as well [11], such as pri-miRNAs coding for peptides [12], interacting with non-Ago proteins [13], activating Toll-like receptors [14], upregulating protein expression, directing transcription, targeting mitochondrial transcripts or nuclear ncRNAs [15]. MiRNAs play a wide role in the life’s processes.

The aim at this study is to investigate the effect of different NFC/NDF levels on the miRNAs participating in rumen development in calves. Rumen tissue was observed by histological sectioning and staining, and high-throughput sequencing was used to identify the miRNAs that affect the rumen development. In addition, the network of regulatory relationships between components of the miRNA-mRNA-cluster network was elucidated by analyzing DEmiRNAs, target genes, and clusters of interest.

## 2. Materials and Methods

### 2.1. Animals and Experimental Design

#### 2.1.1. Ethics Statement

All experiments and animal care procedures were performed in accordance with the protocols and guidelines approved by the Institutional Animal Care and Use Committee (IACUC) of Henan Agriculture University (Zhengzhou, China) (Permit Number: 11-0085; Date: 06-2011).

#### 2.1.2. Experimental Animals and RNA Isolation

Six Charolais hybrid bull calves were used in our study; all calves were raised in the same environmental conditions. Three calves were fed total mixed rations with an NFC/NDF ratio of 1.35 (H group), and three calves were fed total mixed rations with an NFC/NDF ratio of 0.80 (L group). The calves were fed according to the “Chinese Beef cattle Raising Standard” (2004). “Dietary nutrition level (dry basis)” is published in [16]. NDF content in the feed samples was based on the observations by Van Soest [17]. The experiment lasted 105 days, a pre-trial period was 15 days and the trial period was 90 days. Calves were slaughtered at the end of the test. The ventral sac of the rumen was chosen for study because it is the site with the highest capillary blood flow per unit weight mucosa [18]. Rumen tissues were harvested with silver paper then either frozen immediately in liquid nitrogen and stored at −80 °C or prepared for histological sectioning.

#### 2.1.3. Preparation and Observation of Rumen Sections

Rumens were exteriorized and separated as described in Carstens et al. [19]. Rumen tissue was cut into 2 cm^2^ sections with sterile surgical scissors, washed several times in pre-cooled PBS buffer (pH = 7.2), then fixed overnight in 4% paraformaldehyde. Tissue was then dehydrated, cleared, and embedded in paraffin. Samples were cut into 6 μm sections then stained with hematoxylin and eosin (HE) using the standard protocol. The morphological characteristics of the rumen papilla were observed with light microscopy. The papillae length, width, and tunica muscularis were measured five times using Motic images advanced 3.2 software.

### 2.2. MicroRNA Sequencing

#### 2.2.1. miRNA library Construction and Illumina Deep Sequencing

A total of four miRNA libraries were constructed from two rumen tissues per group, using the Illumina^®^ small RNA Library Prep Set (NEB, Ipswich, MA, USA) according to the manufacturer’s protocol. Briefly, 1.5 μg of RNA per sample was brought to 6 µL with H_2_O and adapters were ligated to the 3′ and 5′ ends. These products were used for reverse transcription and amplification. The amplicons were purified by agarose gel separation. The RNA libraries were quantitated using a Qubit 2.0 fluorometer (Life Technologies, Camarillo, CA, USA) and brought to 1 ng/µL. RNA quality was analyzed using an Agilent Bioanalyzer 2100 (Agilent, Santa Clara, CA, USA). The RNA integrity number (RIN) was more than 8. The effective concentration of the miRNA library was assessed using qPCR. The libraries were sequenced using an Illumina HiSeq 2500 system at Biomarker Technologies (Beijing, China).

#### 2.2.2. Sequence Analyses

Raw reads were assessed for quality using the Illumina Pipeline filter (Solexa v0.3). The pipeline performed the following steps: (1) Reads were set aside if more than 20% of their nucleotides had quality scores of less than 30. (2) 3′ adapter sequences were trimmed. (3) Reads were set aside if more than 10% of their nucleotides were unknown (N). (4) Reads shorter than 18 or longer than 30 nucleotides were removed. Each sample yielded more than 19.85 M clean reads. Bowtie v.1.1.0 was used to identify snRNAs, tRNAs, rRNAs, snoRNAs, various ncRNAs, and low-complexity sequences by comparing clean reads against the Repbase, GtRNAdb, Rfam, and Silva databases. Bowtie was also used to map clean reads to the bovine reference genome (UMD_3.1.1). The reads were then compared with the known cattle pre-miRNAs and mature miRNAs in miRBase (v21) [20]. Novel miRNAs were predicted using miRDeep2 [21].

#### 2.2.3. Differential Expression Analysis of miRNAs

To estimate miRNA levels in each sample, data were quantified as transcripts per million clean reads (TPM) to calculate and normalize expression [22]. Differential expression analysis was performed using DESeq R to compare the two groups [23]. MicroRNAs with adjusted *p* ≤ 0.05 and |log2 (fold change)| ≥ 1 were classified as DEmiRNAs.

#### 2.2.4. MiRNA Target Prediction, Functional Annotation, and Interaction Networks

Based on the miRNA sequences, MiRanda [24] and RNAhybrid [25] were used to predict DEmiRNA target genes. Target genes that were recognized by both programs were retained. KOBAS [26] was used to test the statistical enrichment of the target genes in the gene ontology (GO) [27] and Kyoto encyclopedia of genes and genomes (KEGG) [28] databases. Interactions among the miRNAs and mRNAs were constructed and visualized as networks using Cytoscape [29].

### 2.3. Verification of Sequencing Results

#### 2.3.1. Validation of Relative Expression of miRNAs and mRNAs

Seven differentially expressed miRNAs (DEmiRNA) and six target genes were selected. The relative expression levels of five selected DEmiRNAs were randomly validated for the reliability of the sequencing data. The relative expression levels of all of the DEmiRNAs were analyzed by stem-loop quantitative real-time reverse transcription PCR. The predicted relationships between three DEmiRNAs and six target genes were tested. Target genes were validated by quantitative real-time reverse transcription PCR. Total RNA was extracted using TRIzol reagent then reversed transcribed using a PrimeScript RT reagent Kit with gDNA Eraser (TaKaRa). The Specific miRNA RT primers and seven pairs of qPCR primers were designed by RiboBio (RiboBio Co., Guangzhou, Guangdong, China). The mRNA primers (10 μmol/μL) were designed by Biosunya (Biosunya Biotechnology Co. Ltd., Shanghai, China); primers are listed in in Supplementary Table S1. qPCR reactions were performed in triplicate using on a LightCycler 96 instrument (Roche, Indianapolis, IN, USA). The volume of each reaction was 10 µL: 5 µL of SYBR Premix Ex Taq II kit (TaKaRa), 1 µL of a mix of forward and reverse, 3 µL of RNase-free H_2_O, and 1 µL of cDNA. Three common bovine housekeeping genes, glyceraldehyde-3-phosphate dehydrogenase (*GAPDH*), actin beta (*ACTB)*, and beta-2-microglobulin (*B2M*) were tested for being used as internal controls. Since *GAPDH* had the lowest standard deviation (0.64, ± Ct) and a lower coefficient of variation (3.2, %Ct), it was chosen as the internal control standard. U6 snRNA were chosen as the miRNA internal control [30,31,32,33]. The 2^–ΔΔCt^ method was used to determine the relative mRNA and miRNA abundance [34].

#### 2.3.2. Vector Construction

The 3′ untranslated region (UTR) of the *PPARG* gene, containing the bta-miR-128 binding site, was amplified by PCR using bovine genomic DNA as the template. The amplicon was purified then ligated into the XhoI–NotI site of the psiCHECK^TM^-2 vector. The resulting plasmid was used to transform *E. coli* DH5α. Using “white-blue colony selection,” white colonies were cloned then amplified. The final recombinant plasmid was named *PPARG*-3′UTR-WT. The seed region of the bta-miR-128 binding site was mutated (Tsingke Company) and *PPARG*-3′UTR-Mut was constructed. The psiCHECK^TM^-2 reporter plasmid was a gift from Dr. Guirong Sun. Similarly, luciferase vectors of solute carrier family 16 member 1 were constructed (*SLC16A1*-3′UTR-WT and -Mut); *SLC16A1*-3′UTR-Mut was constructed using primer mutation. All plasmids were extracted using an EndoFree Mini Plasmid Kit II (TIANGEN, Beijing, China) and were sequenced by Biosunya Biotechnology Co. Ltd. (Shanghai, China). Primers are listed in Supplementary Table S1.

#### 2.3.3. Cell Culture and Luciferase Reporter Assay

HEK293T [35] cells were maintained in high glucose medium supplemented with 10% fetal bovine serum (Biological Industries, Israel). A total of 5 × 10^5^ cells/well were seeded into each well of a 6-well plate, when approximately 70% confluent, 100 ng of *PPARG*-UTR-WT, and *PPARG*-UTR-Mut were cotransfected with 20 nM negative control (NC) or bta-miR-128 mimic (GenePharma, Shanghai, China) using Lipofectamine 2000 (Solarbio, Beijing, China) according to the manufacturer’s instructions. The medium was replaced after 6 h and the relative luciferase activity was measured after 48 h of using the Dual-Luciferase Reporter Assay System (Solarbio, Beijing, China) on a Fluoskan Ascent FL instrument (Thermo Fisher Scientific, Shanghai, China). *Renilla* luciferase (Rluc) activity was normalized to firefly luciferase activity. Relative luciferase activity was calculated to assess regulation of gene transcription in the treatment group. The experiment was performed using three replicates. Similarly, the target relationship between *SLC16A1* and bta-miR-128 was analyzed.

### 2.4. Statistical Analyses

Data were evaluated for differences by one-way ANOVA using SPSS 18.0 software (IBM, Chicago, IL, USA) * *p* < 0.05; ** *p* < 0.01. Data are expressed as the mean ± standard error of the mean. Origin software (Northampton, MA, USA) and GraphPad Prism 5 software (San Diego, CA, USA) were used for graphics.

## 3. Results and Discussion

### 3.1. Effect of NFC/NDF Levels on Rumen Development of Calf

**Figure 1:**
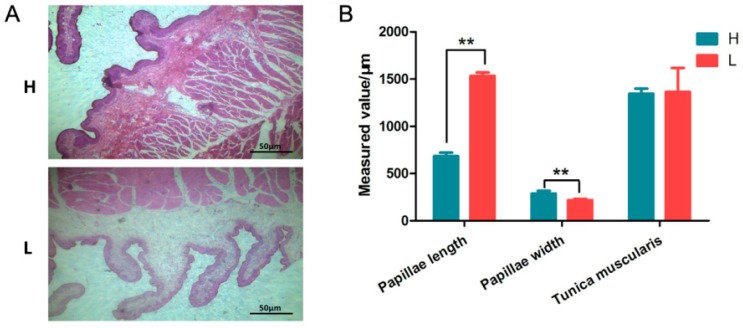
Histological observation of calf rumen tissues (n = 3). (**A**) Histological section of rumen tissues, H and L groups. (**B**) Quantification of papillae length and width, and the thickness of the tunica muscularis.

The rumen is a digestive organ unique to ruminant animals (sheep, cattle, goats, deer, giraffes, and llamas). The development of its epithelium, particularly the height of the papillae, greatly affects the digestive function [36,37]. Growth and development of rumen epithelium is influenced by numerous factors. Steele, M.A et al. have reported that dietary energy levels affect the morphological development of rumen epithelium, and a diet high in grain damages the epithelium in cattle [38]. In this study, Charolais hybrid bull calves were fed a high and low ratio of NFC/NDF, the H group was fed a ratio of NFC/NDF of 1.35, and L group was fed a ratio of NFC/NDF of 0.80. After 105 days, samples of the rumen tissue were collected. The evidence from HE staining revealed that papillae length in the H group was significantly shorter than that in the L group (*p* < 0.05); however, the papillae width of H group was significantly wider than in the L group (*p* < 0.05) (Figure 1A,B). We also observed no obvious difference in the thickness of the tunica muscularis between the groups. This result suggested that the level of NFC/NDF affects the development of papillae particularly their length and width. The molecular mechanism behind how NFC/NDF levels influence rumen development is not well understood, so to gain a better understanding, at the molecular level, of the effect of NFC/NDF levels in these calves, the miRNA profiles of the tissues were compared.

### 3.2. Overview of Small RNA Deep Sequencing Data

**Figure 2:**
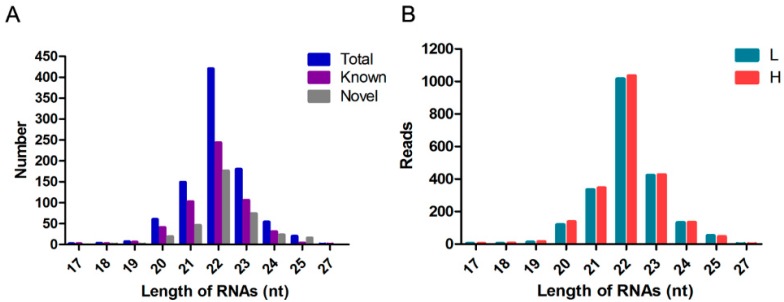
Summary of miRNA-seq data. (**A**) Distribution of miRNA sequences lengths (total, known, and novel). (**B**) Distribution of miRNA sequence reads between the H and L groups.

Using the Illumina HiSeq 2500 platform, four libraries from two groups were constructed and sequenced, yielding more than 1.48 million reads ranging in length from 18–30 nt (Table S2). Over 90% of the reads were retained after quality control and were analyzed to identify the candidate miRNAs (Table S2). A total of 896 miRNAs were found, of which 540 had been identified previously, and 356 were novel predicted miRNAs. The distribution of the lengths of the mature miRNAs is presented in Figure 2A. The most common length was 21–23 nt (Figure 2A), and the length distributions of the two groups appear to be identical (Figure 2B). These results suggest that the methods used in this study reliably identified the miRNAs.

### 3.3. Identification of Differentially Expressed miRNAs

**Figure 3:**
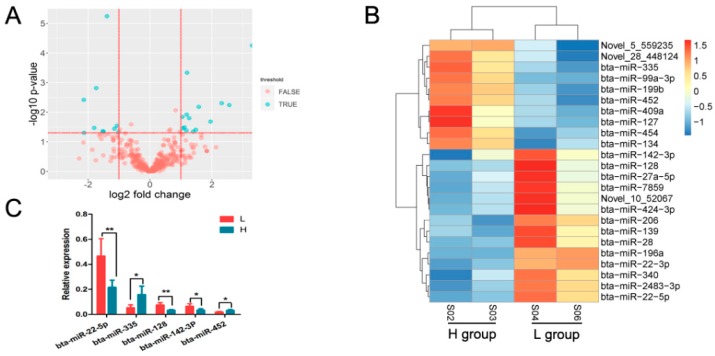
Overview of miRNA deep sequencing data. (**A**) Volcano plot for differentially expressed miRNAs. (**B**) Heatmap plot for relative expression abundance of DEmiRNAs. (**C**) Validation of DEmiRNAs by qPCR.

Of the 24 DEmiRNAs identified between the groups, 14 were up-regulated and 10 were down-regulated in the L group relative to the H group (Figure 3A), and of the 24, three were novel miRNAs (Novel_28_448124, Novel_10_52067 and Novel_5_559235) (Table S3). Figure 3B shows the results of a clustering analysis based on the expression profiles for the 24 DEmiRNAs. To validate the expression levels, five DEmiRNAs were selected and their abundance was measured using qPCR. The results were consistent with those obtained from miRNA deep sequencing (Figure 3C). Among the DEmiRNAs, expression of bta-miR-199b was higher in rumen tissue (Table S3). Studies on rumen development in calves indicate that abundance of bta-miR-199b and bta-miR-22-3p change in opposite directions before and after weaning, which is in agreement with the present study [4]. miR-128 [39], miR-127 [40], miR-134 [41], and miR-139 [42] have also been reported to influence the cell proliferation and apoptosis.

### 3.4. Prediction of DEmiRNA Target Genes

**Figure 4:**
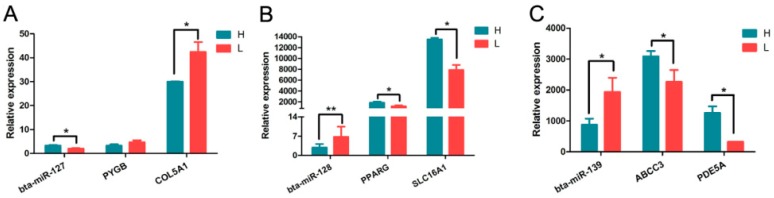
Validation the expression relationship between three DEmiRNAs and target genes by qPCR. (**A**) Relative expression of bta-miR-127 and target genes *PYGB* and *COL5A1*. (**B**) Relative expression of bta-miR-128 and target genes *PPARG* and *SLC16A1*. (**C**) Relative expression of bta-miR-139 and target genes *ABCC3* and *PDE5A*.

To characterize the regulatory roles of the miRNAs in rumen growth and development, target genes were predicted for the DEmiRNAs, resulting in 243 potential target genes. bta-miR-127 was associated with the most target genes, followed by bta-miR-139, bta-miR-27a-5p, and bta-miR-134 (Table S3). miRNAs usually suppress protein-encoding mRNAs by complementary binding to the 3′UTR [43]. To validate the negative regulatory relationship between miRNAs and their targets, three DEmiRNAs and six target genes (bta-miR-127 with target genes *PYGB/COL5A1*, bta-miR-128 with target genes *PPARG*/*SLC16A1*, and bta-miR-139 with target genes *ABCC3*/*PDE5A*) were investigated (Figure 4A–C and Table S4). A negative correlation in expression levels was observed between all miRNAs and their targets. Significant differences were identified between groups, except for the *PYGB* gene. This suggests that the predicted miRNA-target relationships have been verified in this study.

### 3.5. Functional Enrichment Analysis

**Figure 5:**
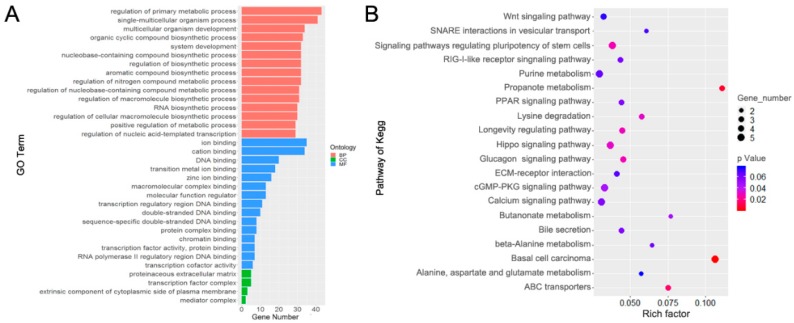
Functional enrichment analyses of DEmiRNAs. (**A**) Gene ontology (GO) enrichment analysis of target genes. Biological process (BP), cellular component (CC), molecular function (MF). (**B**) Kyoto encyclopedia of genes and genomes (KEGG) pathways enrichment analysis of target genes.

**Figure 6:**
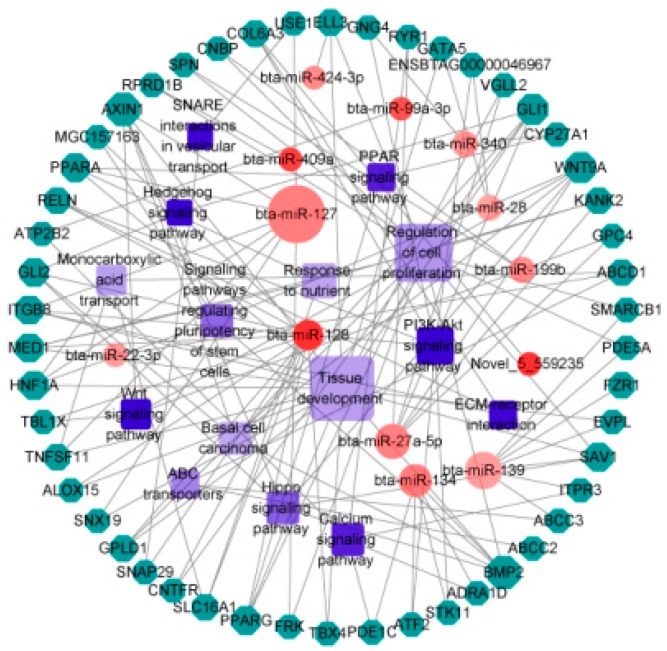
Analysis of miRNA-mRNA-cluster network among DEmiRNAs, target genes, and clusters of interest. Circles represent DEmiRNAs, octagons represent genes, and squares represent clusters. The size of the shape indicates the number of targets. The deeper the color of the red circle, the greater the absolute value of log_2_FC, and the deeper the color of the blue square, the greater the *p* value.

Functional enrichment analysis revealed target genes mainly enriched in regulation of primary metabolic process, single-multicellular organism process, ion binding, DNA binding, and proteinaceous extracellular matrix (Figure 5A and Table S5). The significantly enriched pathways included basal cell carcinoma, ABC transporters, hippo signaling pathway, and calcium signaling pathway (Figure 5B). To better understand the function of the genes of interest in rumen epithelium development, the relationships amongst miRNAs, target genes, and clusters were visualized as an integrated network (Figure 6 and Table S7). The network included DEmiRNAs such as bta-miR-127, bta-miR-128, and bta-miR-139, and their target genes. The possible functional role in rumen development of the clusters is discussed below.

The rumen epithelium is keratinized stratified squamous epithelium, including the basal layer, the spinous layer, the granular layer, and the stratum corneum [44]. Early studies showed that dietary energy level affects the ruminal epithelium development, and that a diet high in grain induces epithelium damage and keratosis in cattle [45,46]. Levels of NFC/NDF, nutrient composition, and fiber content affect the rumen environment, playing an important role in the chemical and physical stimuli of the rumen epithelium. Therefore, cell proliferation, differentiation, tissue development, and nutrient and physical stimuli related clusters are noteworthy.

Not unexpectedly, many target genes are clustered in cell proliferation and tissue development. The genes in these clusters are involved in basal cell carcinoma, Hedgehog signaling pathway, Wnt signaling pathway, PI3K-Akt signaling pathway, and signaling pathways regulating pluripotency of stem cells (Figure 6). These pathways play an important role in the basal cell carcinoma and cell proliferation [47]. Both the up-regulation of GLI family zinc finger 2 (*GLI2*) expression in the Hedgehog pathway and the activation of β-catenin signal in the Wnt pathway function in cell proliferation and the occurrence of basal cell carcinoma [48]. Many genes were also clustered in extracellular matrix (ECM)-receptor interaction and the Hippo signaling pathway. The Hippo signaling pathway is involved in the growth of mammalian tissues, regulating cell proliferation, and programmed death [49]. Extracellular matrix is secreted by epithelial cells; the epithelial cells secrete collagen and membrane mucin on the basal layer of the epithelial tissue. These proteins act as signals directing epithelial cell growth and migration. Epithelial cells develop along the basal layer during embryonic development or callus regeneration [50]. These genes or pathways may play a crucial role in rumen epithelium growth and affect the papilla growth and development.

The calcium signaling and the peroxisome proliferator activated receptor (*PPAR*) signaling pathways were enriched by many target genes. These pathways are involved in cell proliferation, apoptosis, cytokeratosis, and cell repair process. However excessive thickness of the cuticle reduces volatile fatty acid absorption and rumen epithelial blood flow [51]. The growth and regeneration of the rumen epithelium directly affects the absorption and transport of nutrients and ultimately the growth and health of the animal [36]. The thickness of the keratinous layer in the rumens of the high concentrate group was significantly lower than those in the low concentrate group [52]. This has been classically illustrated by studies showing that increasing the calcium concentration in the culture medium causes terminal differentiation of primary keratinocytes and inhibition of DNA synthesis [53]. The enriched genes, phosphodiesterase 1C (*PDE1C*) [54], inositol 1,4,5-trisphosphate receptor type 3 (*ITPR3*) [55], ATPase plasma membrane Ca^2+^ transporting 2 (*ATP2B2*) [56], and ryanodine receptor 1 (*RYR1*) [57], function in the calcium signaling pathway and have a role in cell proliferation and death [58,59,60,61] (Table S6). *PPARG* is one of the members of the *PPARs* superfamily [62]. Activation of *PPARG* ligands induced terminal differentiation and apoptosis of keratinocytes and apoptosis in a variety of cell types, including epithelial cancer cell lines [63].

A low NFC/NDF diet is characterized by high fiber and low nutrient levels. Greater amounts of fiber generate more stimulus in rumen epithelium, and this stimulation promotes rumen development [64]. In this study, we found some target genes were enriched in the regulation of extracellular stimuli, namely the KN motif and ankyrin repeat domains 2 (*KANK2*), mediator complex subunit 1 (*MED1*), and peroxisome proliferator activated receptor alpha (*PPARA*) (Figure 6 and Table S5). A high grain content diet can inhibit the growth of rumen papillae because of the lower fiber content of grain. This is consistent with the results of our study, calves fed a diet with less neutral detergent fiber (NDF) had shorter rumen papillae (H group) than calves fed a diet higher in NDF (L group) (Figure 1A). Therefore, the physical stimuli from dietary fiber affects rumen papilla to some extent.

The transport of nutrients is essential for the growth and development of the cells. In this study, some of the target genes were associated with nutritional response cluster, such as *KANK2*, solute carrier family 16 member 1 (*SLC16A1*), and *MED1* (Figure 6 and Table S5). We also found target genes involved in monocarboxylic acid transport, such as *SLC16A1*, TNF superfamily member 11 (*TNFSF11*), HNF1 homeobox A (*HNF1A*), and ATP binding cassette subfamily D member 1 (*ABCD1*) (Figure 6 and Table S5). A large number of studies have shown that short-chain fatty acids produced by fermentation in the rumen are absorbed by rumen cells and metabolizes ketones or lactic acid within the cells or as a source of energy for epithelial cells and most of them were transported to portal vein blood by monocarboxylic acid transporter (*MCT*) [65,66,67]. *MCT1* (*SLC16A1*) is located at the base of the basal cells in cattle and sheep [68]. The general role of *MCT1* is to take up or release lactic acid from hypoxic-exposed cells, in order to maintain lactate levels during of glycolysis, gluconeogenesis, and lipogenesis [67,69]. Lactic acid can reactivate the tumor microenvironment providing energy for the tumor and thereby promoting its growth [70,71]. In this study, we observed that the relative expression of *SLC16A1* in L group was significantly lower than in the H group (Figure 4B). The accumulation of lactic acid in rumen epithelial cells might promote the development of papillae.

### 3.6. Targeting Effect of Bta-miR-128 on PPARG and SLC16A1

**Figure 7:**
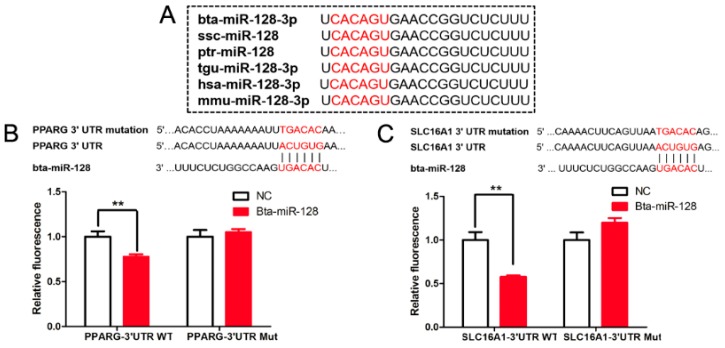
Regulatory effect of bta-miR-128 on PPARG and SLC16A1. (**A**) Alignment of mature miR-128 sequences from various species. (**B**) Inhibitory effect of bta-miR-128 on *PPARG* 3′UTR, using the dual-luciferase system. Target site of bta-miR-128 on *PPARG* mRNA 3′UTR and its mutant variant (above) (** *p* < 0.01). (**C**) Inhibitory effect of bta-miR-128 on *SLC16A1* 3′UTR, using the dual-luciferase system. Target site of bta-miR-128 on *SLC16A1* 3′UTR and its mutant variant (above) (** *p* < 0.01). WT, wild type; Mut, mutant type.

We focused on bta-miR-128, which was upregulated in the L group, for a deeper exploration of the biological significance of a candidate DEmiRNA. The mature miR-128 sequence is highly conserved among the various species including pig, zebra finch, human, and mouse (Figure 7A). To identify the direct binding site of miR-128 on *PPARG* and *SLC16A1*, a 3′ UTR fragment of the putative targeting sites with a seed region binding site was inserted into the psiCHECK-2 vector (Figure 7B,C). Luciferase assay revealed that bta-miR-128 significantly reduced the Rluc activity of the wild-type *PPARG* and *SLC16A1* reporter vector, while point mutations of the seed region of bta-miR-128 disrupted the suppression (Figure 7B,C). Taken together, these data indicated a negative regulatory relationship between bta-miR-128 and *PPARG* and *SLC16A1*. *PPARG* and *SLC16A1* are, respectively, involved in cell differentiation and nutritional response processes in the rumen thereby influencing the rumen development. We conclude that miR-128 is an important miRNA functioning in the rumen development and is affected by NFC/NDF levels.

## 4. Conclusions

In summary, we observed obvious differences in papillae length and width between the rumens of calves fed a diet with high NFC/NDF vs. those fed low NFC/NDF. We have constructed a miRNA-mRNA-cluster network and found through cluster network analysis that cell proliferation, differentiation, physical and nutrient stimuli processes participate in rumen development. In addition, our results suggest that bta-miR-128 is controlled by NFC/NDF levels and may influence the rumen development via regulating PPARG and SLC16A1 expression. Our findings provided evidence for studying the rumen development and absorption.

## Supplementary Materials

The following are available online at https://www.mdpi.com/2076-2615/9/8/496/s1, Table S1: List of cloning and qPCR primers. Table S2: Descriptive summary of miRNAs reads. Table S3: List of the DEmiRNAs identified between H and L rumen tissues. Table S4: Information on target genes of DEmiRNAs. Table S5: List of target genes of DEmiRNAs enriched in GO categories. Table S6: List of target genes of DEmiRNAs enriched in KEGG pathway. Table S7: List of the miRNA-mRNA-cluster network among DEmiRNAs, target genes and clusters of interest.
